# Survivability and behavior of probiotic bacteria encapsulated by internal gelation in non-dairy matrix and *In Vitro* GIT conditions

**DOI:** 10.1371/journal.pone.0303091

**Published:** 2024-06-21

**Authors:** Safdar Nazir, Muhammad Afzaal, Farhan Saeed, Aftab Ahmad, Huda Ateeq, Ali Ikram, Amara Rasheed, Faiza Kiran, Noor Akram, Faiyaz Ahmed, Aasma Asghar, Mahbubur Rahman Khan

**Affiliations:** 1 Department of Food Science, Government College University Faisalabad, Faisalabad, Pakistan; 2 Department of Food Science, Food Safety & Biotechnology Lab, Government College University Faisalabad, Faisalabad, Pakistan; 3 Department of Nutritional Sciences, Government College University Faisalabad, Faisalabad, Pakistan; 4 University Institute of Food Science and Technology, The University of Lahore, Lahore, Pakistan; 5 Department of Basic Health Sciences, College of Applied Medical Sciences, Qassim University, Buraydah, Saudi Arabia; 6 Department of Food Processing and Preservation, Hajee Mohammad Danesh Science & Technology University, Dinajpur, Bangladesh; Universidad Autonoma de Chihuahua, MEXICO

## Abstract

The primary objective of this investigation was to assess the viability of free and encapsulated *Lactobacillus plantarum* probiotics in mango juice and under simulated gastrointestinal conditions. Specifically, the probiotics were encapsulated using sodium alginate and alginate-soy protein isolate through the internal gelation method, and the obtained probiotics were characterized for various attributes. Both free and encapsulated probiotics were exposed to challenging conditions, including thermal stress, low temperature, and simulated gastrointestinal conditions. Additionally, both types of probiotics were incorporated into mango juice, and their survival was monitored over a 28-day storage period. Following viability under simulated gastrointestinal conditions, the count of free and encapsulated probiotic cells decreased from initial levels of 9.57 log CFU/mL, 9.55 log CFU/mL, and 9.53 log CFU/mL, 9.56 log CFU/mL to final levels of 6.14 log CFU/mL, 8.31 log CFU/mL, and 6.24 log CFU/mL, 8.62 log CFU/mL, respectively. Notably, encapsulated probiotics exhibited a decrease of 1.24 log CFU and 0.94 log CFU, while free cells experienced a reduction of 3.43 log CFU and 6.24 log CFU in mango juice over the storage period. Encapsulated probiotics demonstrated higher viability in mango juice compared to free probiotics throughout the 28-day storage period. These findings suggest that mango juice can be enriched with probiotics to create a health-promoting beverage.

## 1. Introduction

When given to a host in sufficient quantities, live microorganisms with favorable physiological effects are known as probiotics. A group of functional foods, well-known as probiotic foods, involves significant commercial interests and a growing market share [1]. Food manufacturers are attracted to probiotics due to the anticipated market growth, healthy margins, and growing consumer interest in functional foods. Additionally, probiotics have been associated with better immune function, reduced risk of allergies and cancer, better digestive health, the avoidance of urogenital infections, and lower blood pressure and cholesterol levels [2]. In the previous ten years, the market has seen the emergence of more than 500 novel probiotic fortified foods and beverages. The scientific community frequently considers 10^8^ to 10^9^ CFU/g (Colony Forming Units per gram) of probiotic viable cells consumed daily as the least necessary to produce the desired effects [3]. Moreover, a 100 mL portion of the tropical mango juice containing *L*. *rhamnosus* or *L*. *plantarum* carried at least 9.96 log CFU/mL or 9.74 log CFU/mL (colony-forming unit per milliliter) of these microorganisms [4]. Today, a wide range of probiotic products, mainly dairy, is on the market, and more are yet to come, especially non-dairy novelties. The maintenance of cell viability in probiotic-containing products is still considerably challenging. The microorganisms must survive during intestinal transit and harsh environmental conditions, which is often detrimental to the cell viability of the final product [5]. Encapsulation of probiotics has been shown to protect probiotics from damaging environmental factors. Microencapsulation can also improve the survival of probiotic cells during gastrointestinal digestion by protecting them from the stomach’s acidic environment. In this context, the encapsulation of probiotics could provide the required protection to the administrated bacterial cells and, thus, increase the delivered amount [6]. To successfully encapsulate viable cells into foodstuff, it is paramount to preserve the bacterial viability in all of the manipulation procedures and choose accordingly the encapsulation materials, which must be food compatible [6].

Most adopted encapsulation materials are alginate-based and protein-based materials. Soybean protein isolate (SPI) is produced from soybean meal and sodium alginate (SA) is a linear polysaccharide obtained from seaweed. Sodium alginate (SA), is being extensively used to encapsulate probiotics due to its excellent pH-responsive properties [7]. Therefore, in the current study sodium alginate (SA) and Soy protein isolate (SPI) have been used as they possess excellent encapsulation efficiency. Encapsulation by internal gelation through alginate encapsulating material may be the appropriate methods for encapsulating probiotics [8]. Mango (*Mangifera indica L*.) is the king among tropical fruits globally and is currently ranked 5th in total world production among the major fruit crops [9]. Mango is relished for its juiciness, exotic flavor, delicious taste, and high nutritional value. Mango is a nutrient‐rich fruit containing carbohydrates, dietary fiber (pectin), vitamin C, vitamin A (from β‐carotene), and many other phytochemicals for maintaining normal health [10]. Mango juice can be considered an appropriate carrier for the transmission of probiotics in the form of microencapsulated beads because of the fruit’s nutritional profile and phytochemical content [11]. This study aims to investigate the survivability of *Lactobacillus plantarum* probiotics, both free and encapsulated, in mango juice and under simulated gastrointestinal conditions.

## 2. Materials and methods

The research was conducted at Food Safety and Biotechnology Laboratory, Department of Food science, Government College University Faisalabad. In the current study, the necessary media and chemicals were purchased from Merck (Germany) and Sigma Aldrich. Encapsulating material and glassware were purchased from the local scientific stores of Faisalabad. Raw mangoes (variety Chaunsa) were purchased from Jhang bazar, Faisalabad. *L*. *plantarum* was received in a pure culture from the University of Agriculture Faisalabad, in freeze-dried form.

### 2.1. Bacteria culture preparation

Activation of bacterial culture (*L*. *plantarum*) was performed as previously done by Afzaal *et al*. [12], briefly, MRS agar method was used for the activation of lactic acid bacteria, briefly, 100ml of MRS agar medium was prepared by dissolving the MRS agar powder in distilled water and sterilized by autoclave. Medium was allowed to cool at 45–50°C before pouring into sterile Petri dishes. Bacterial culture was spread evenly over the surface of the agar, the MRS agar plates inoculated with the bacterial culture by using a sterile loop or pipette. The plates were incubated at 30–37°C as this was appropriate temperature to culture lactic acid bacteria. Colonies started to appear within 24 hours. The cells were harvested and weigh in, data was recorded. The cells concentration was adjusted at 10^10^ CFU/mL.

### 2.2. Preparation of hydrogel solution

Alginate-soy protein isolate-based hydrogel beads were produced by the gelation method as described by Praepanitchai *et al*. [13], with slight modifications. The biopolymer solutions of 4% (w/v) sodium alginate and 20% (w/v) of soy protein isolate proteins were prepared in distilled water. Various ratios of homogenous aqueous solutions of alginate and soy protein isolate were prepared as shown in Table 1, that were used as wall materials to encapsulate probiotic cells.

**Table 1:** Preparation of hydrogels (SA-SPI) with different ratios.

Formulations	F1	F2	F3	F4
Ratio (SA:SPI)	2:0	2:2	2:4	2:6

*SA = Sodium alginate,

*SPI = Soy Protein Isolate

### 2.3. Preparation of beads

The beads for microencapsulation were prepared by following the process Afzaal *et al*. [8]. Briefly, 5 ml of SA and SPI, with different rations as mentioned in Table 1, was taken in a 5 ml syringe and poured in a beaker containing germ-free oil and 5 g of tween-80 solution as an emulsifier, the solution obtained with both encapsulating materials was then dispersed, and the mixture was stirred with the help of a magnetic stirrer at 150 rpm. In general, Calcium chloride was added dropwise until the emulsion broke. The prepared hydrogel beads were washed twice with saline solution (0.9%w/v) and stored at 4^°^C for further use. The purpose of using different ratios was to evaluate the comparison of survival and viability of probiotics in different ratios.

### 2.4. Encapsulation efficiency

The encapsulation efficiency of the probiotic cells in hydrogel beads of alginate and alginate-soy protein isolate was determined with slight modification by the method described by Zeashan *et al*. [14], briefly, probiotic cells loaded with hydrogel beads (1 g) were then centrifuged at 6000 rpm with the help of centrifuge machine (Centrikon T-324, Kontron Instrument, Germany) in 9mL of phosphate buffer saline solution (0.1MPBS; pH 7.4) at 4° C for 24 h suspension for 30 min. The cell pellets were plate-counted, results were taken in triplicates and the encapsulation efficiency of probiotic cells (CFU) was determined as follows:

$$Encapsulation\;efficiency\;(\%)=\left(\frac{cells\;count\;(CFU/mL)\;after\;disintegration\;of\;the\;hydrogel\;beads}{Initial\;loading\;of\;the\;cells\;(CFU/mL)\;in\;hydrogel\;beads}\times 100\right)$$

### 2.5. Viability under heat treatment

The viability of *L*. *plantarum*-encapsulated hydrogel beads dispersed in normal saline solution (0.9% w/v) was exposed to heat treatment following the method described by Tiani *et al*. [15] with slight modifications. Free cell and *L*. *plantarum*-encapsulated beads (10^10^ CFU/ml) were placed in test tubes containing 9 mL of normal saline solution (0.9% w/v). The test tubes were further incubated in a water bath at various temperatures (30, 50, 63, and 72^°^C) for 5 min. The samples were cooled down to room temperature (∼25^°^C). The viability of the free and encapsulated *L*. *plantarum* probiotics was obtained.

### 2.6. Survival of free and encapsulated probiotics at refrigeration temperature

To analyze probiotic resistance under refrigeration conditions the method as previously followed by Brinques and Ayub [16] was used however, temperature was slightly modified in the process. The viability of *L*. *plantarum* under refrigeration was evaluated by incubating 0.4 mL (approximately 9.5 log CFU/g) of free or immobilized hydrogel beads in 1.8 mL of sterile sodium chloride solution (0.5%, w/v) and kept in the refrigerator at 4°C. Aliquots of 1 mL were taken every other day for 28 days to determine the total number of viable cells. The immobilized beads were dissolved in the appropriate Phosphate buffer solution (PBS) and used to determine the total number of viable cells. The viability count was calculated by following formula:

Viability(%)=(Initialnumberofviablecells/Numberofviablecellsatspecifictimepoint)×100

### 2.7. Viability under Simulated gastrointestinal conditions (SGIC)

Viability analysis of probiotics under simulated gastrointestinal conditions is important to determine the ability of probiotic strains to survive and colonize the human digestive system, which is a critical factor in their efficacy. To access the viability of free and encapsulated strains under simulated gastrointestinal conditions procedure outlined by Varela-Pérez *et al*. [17] was followed with slight modifications. Simulated gastric fluid (SGF) was prepared by dissolving 3 g of pepsin and 6 g of sodium chloride in 1000 ml of deionized water, pH was adjusted to 3 by adding hydrochloric acid (HCl). On the other hand, simulated intestinal fluid (SIF) was prepared by dissolving 6.8 g monobasic potassium phosphate, 13.6 g dibasic potassium phosphate and 6.8 g of sodium chloride (NaCl) in 1000 ml deionized water, whereas pH was adjusted at 6.8 by adding Potassium hydroxide (KOH). Prepared samples were exposed to both fluids, SGF & SIF in which pH of the solutions was already adjusted. A membrane filter of 0.22 µm was used to add pepsin solution into the bacterial solutions up to the concentration of 1000 units/mL, this step was followed by addition of a solution of filter-sterilized bile salts at 0.3% (w/v). Gastric fluid-bacterial solutions were incubated for 2 hours for gastric simulation under anaerobic conditions, whereas intestinal fluid-bacterial solutions were incubated for 3 hours under anaerobic conditions for intestinal simulation. The experiments were triplicated for strains at both states (free and encapsulated). Individual colonies formed were counted in terms of colony forming units as per milliliter of the sample (CFU/mL) and the results were expressed as Log^10^ values. The viability count was calculated by following formula:

Viability(%)=(Initialnumberofviablecells/Numberofviablecellsatspecifictimepoint)×100

### 2.8. Pasteurized mango juice preparation

The pasteurized mango juice was prepared by following the method of Praepanitchai *et al*. [13] with a little modification. Mangoes were appropriately washed and sliced into cubes. Afterward, the slices were blended with the addition of water and sugar and pasteurized at 65°C for 20 min. After the preparation of mango juice, the next step is the probiotic inoculation. 6 jars/bottles containing 1 liter of mango juice were mixed with different probiotic concentrations of both free and encapsulated. The product development plan for the juice is discussed in Table 2. The pasteurized mango juice was incubated at 37°C for 12 h and stored at 4°C for 28 days for physicochemical and microbial analysis. The samples for consumer acceptance were stored at 4°C in sterile plastic and glass bottles for three days before analysis was conducted.

**Table 2:** Product development plan.

Treatment	Description
M_1_	Control
M_2_	Free probiotics
M_3_	Incorporated with F_1_, SA: SPI (2:0)
M_4_	Incorporated with F_2_, SA: SPI (2:2)
M_5_	Incorporated with F_3_, SA: SPI (2:4)
M_6_	Incorporated with F_4_, SA: SPI (2:6)

### 2.9. Physicochemical analysis of mango juice

#### 2.9.1. pH

pH of pasteurized mango juice treatments was measured by using a digital pH meter. The Calibration of the pH meter was done using buffer solutions. The samples were taken in a small beaker mixed thoroughly and the electrode was dipped to note the reading. Measurements were done in triplicate.

#### 2.9.2. Acidity

Acidity was determined by the method described by Kulla *et al*. [18]. Pasteurized mango juice treatments were taken in a small beaker. A few drops of phenolphthalein indicator solution were added dropwise. The sample was titrated against N/10 NaOH until a slight pink colour appeared. Acidity was calculated by using the formula below.


$$Acidity\;\%=\frac{0.009\times N/10\;NaOH\;used\;(mL)}{Wt.of\;the\;sample\;(g)}\times 100$$

#### 2.9.3. Brix

The Brix of pasteurized mango juice treatments was determined by using the refractometer, as the method described by Kulla *et al*. [18].

### 2.10. Microbiological analysis

#### 2.10.1. Probiotic viability

The viaility of probiotics in pasteurized mango juice treatments was determined as described by Vinderola *et al*. [19]. Spread plating method was used in the procedure, the petri plates were incubated and colonies were counted after incubation of 48 hours. Pasteurized mango juice treatments were stored at 4^°^C and was analyzed with an interval of 0, 7, 14, 21, 28 and 35 days.

#### 2.10.2. *E*.*coli* count

Coliform Count in all pasteurized mango juice treatments was determined as described in APHA Standard methods for the examination of dairy products (1992). To determine the *E*. *coli* count in juice, a specific method involving dilution steps was employed. The juice sample was initially diluted in a sterile diluent solution. Common dilution factors used in microbiological analysis range from 10^-1 to 10^-5. For instance, a 1:10 dilution (10^-1) implies mixing 1 part of the juice sample with 9 parts of diluent, while a 1:100 dilutions (10^-2) involves mixing 1 part of the 10^-1 dilution with 9 parts of diluent. After dilution, a measured volume of the appropriately diluted juice sample was spread or plated onto a selective agar medium suitable for *E*. *coli* growth, such as MacConkey agar. This step is crucial for isolating and enumerating *E*. *coli* colonies. The agar plates were then incubated at an appropriate temperature (usually 37°C) for a specified period, typically 24 hours.

### 2.11. Sensory analysis

Mango juice samples for sensory evaluation were presented to the sensory panelist (faculty, MS and PhD students). They were first presented with the attributes of mango juice, after which their remarks were recorded on a 9-point hedonic scale. Participants were asked to evaluate the pasteurized mango juice based on overall liking, colour, flavour, mouthfeel and texture using a 9-point hedonic scale: 1 = dislike extremely and 9 = like extremely [20].

### 2.12. Statistical analysis

The data was statistically analyzed with appropriate model of spss (version 2.0). The collected data was statistically analyzed through mean values and standard deviation in order to evaluate the statistical significance of every parameter. Moreover, the level of significance was chosen as p<0.05 and one-way ANOVA was performed.

## 3. Results and discussion

### 3.1. Encapsulation yield

The final probiotic levels in carrier food are influenced by the encapsulation efficiency, which is crucial for achieving the recommended probiotic concentration. The effectiveness of encapsulation is dependent on the characteristics of hydrogel materials. The materials, SA: SPI (sodium alginate: soy protein isolate), serve as continuous polymers, forming a protective barrier between probiotics and the external environment. Encapsulation yield, as shown in Table 3, illustrates the initial probiotic count before and after encapsulation. Notably, the investigation reveals that SA: SPI (2:4) starch microcapsules exhibit high efficacy. Similar results were reported by Praepanitchai *et al*. [13], indicating comparable yield percentages for viable cells when encapsulating probiotic cells.

**Table 3:** Encapsulation yield (the initial count before and after encapsulation).

Beads	Initial count (before encapsulation) (Log CFU/g)	Final count (after encapsulation) (Log CFU/g)	% efficiency
**F**_**1**_	9.55±0.34	8.95±0.02	**93.72**
**F**_**2**_	9.54±0.27	8.23±0.01	**86.27**
**F**_**3**_	9.56±0.21	9.17±0.06	**95.92**
**F**_**4**_	9.55±0.28	8.54±0.01	**89.43**

F_1_ **=** Encapsulated with SA: SPI (2:0)

F_2_ = Encapsulated with SA: SPI (2:2)

F_3_ = Encapsulated with SA: SPI (2:4)

F_4_ = Encapsulated with SA: SPI (2:6)

### 3.2. Analysis of hydrogel beads under heat treatment

The average outcomes from Table 4, depicting the interactions of treatments (F_1_, F_2_, F_3_, and F_4_) with different storage temperatures (63, 65, and 72°C), revealed that the highest heat tolerance effect was observed in F_2_ (9.70) at 63°C, while the lowest was recorded in F_4_ (6.44) at 72°C. Notably, microencapsulated probiotic bacteria exhibited robust survival at 63°C for 30 minutes, proving to be lethal. Consequently, the study suggests that microencapsulation enhances the survival rate of probiotics in mango juice. These findings align with Mansouripour *et al*. [21] research, where encapsulated bacteria exhibited increased survival, with the D-value doubling at 65°C for 30 minutes. Moreover, in another study reported by Praepanitchai *et al*. [13] hybrid hydrogel beads composed of alginate and soy protein isolate were created and assessed to improve the survival of encapsulated probiotics, specifically *Lactobacillus plantarum*, during the heat processing stage for incorporation into mango juice. The hybrid hydrogel beads demonstrated higher survival rates for the probiotic cells.

**Table 4:** Mean table showing the effect of hydrogel microbeads under heat treatment.

Treatments	63° C	65° C	72° C
**F**_**1**_	9.51±0.4	8.85±1.2	7.33±0.8
**F**_**2**_	9.70±0.7	9.31±1.5	8.11±1.8
**F**_**3**_	9.60±0.3	9.57±0.4	8.84±1.4
**F**_**4**_	9.30±0.8	8.72±0.6	6.43±0.3

F_1_ **=** Encapsulated with SA: SPI (2:0)

F_2_ = Encapsulated with SA: SPI (2:2)

F_3_ = Encapsulated with SA: SPI (2:4)

F_4_ = Encapsulated with SA: SPI (2:6)

### 3.3. Refrigeration storage analysis

The highest observed impact of refrigeration storage was recorded in treatment F_3_ (9.59) with no storage days, while the lowest value was found in treatment F_2_ (6.19) after 28 days of storage. These findings, derived from the mean results of the interaction between treatments (F_1_, F_2_, F_3_, and F_4_) and storage temperatures (63°C, 65°C, and 72°C) are summarized in Table 5. The study results indicate that combining SA and SPI in a 2:4 ratio enhances the survival capability of probiotic bacteria, particularly under low refrigeration temperatures. A recent investigation by Peredo *et al*. [22] explored the impact of storage temperature (4 and 22°C) over different time intervals on the viability of encapsulated bacteria (expressed as log CFU g-1). Additionally, Brinques and Ayub [16] proposed encapsulation as a promising strategy to enhance the viability of probiotics in formulated products.

**Table 5:** Mean table showing the effect of hydrogel microbeads under refrigeration storage.

Treatments	0 day	7 day	14 day	21 day	28 day
**F**_**1**_	9.58±0.6	9.15±0.2	8.34±0.6	**7.26**±0.5	**6.23**±0.8
**F**_**2**_	8.55±0.7	9.11±1.1	8.45±0.4	**7.31**±0.9	**6.19**±0.4
**F**_**3**_	9.59±0.9	9.24±1.6	8.91±1.4	**8.22**±1.4	**7.69**±1.4
**F**_**4**_	9.51±0.4	8.98±0.7	8.17±0.8	**7.92**±0.5	**7.48**±0.6

F_1_ **=** Encapsulated with SA: SPI (2:0)

F_2_ = Encapsulated with SA: SPI (2:2)

F_3_ = Encapsulated with SA: SPI (2:4)

F_4_ = Encapsulated with SA: SPI (2:6)

According to Zhang et al. [23], mixture of alginate and soy protein isolate was developed as a wall material to encapsulate, *Enterococcus faecalis* HZNU P2 (*E*. *faecalis* HZNU P2). The viability of *E*. *faecalis* HZNU P2, when encapsulated and stored for two weeks at 4°C, was completely maintained.

### 3.4. Viability under simulated conditions

#### 3.4.1. Survival of encapsulated probiotic hydrogel microbeads in gastric Fluid (GF)

The mean square outcomes (Table 6) for simulated gastric conditions across all treatments, including those encapsulated with alginate and alginate-soy protein isolate, revealed that the highest reading for simulated gastric conditions (9.00) was observed in treatment F_3_ (SA: SPI 2:4), while the lowest value (7.91) was recorded in F_1_ (SA, 2:0). Analyzing the mean results for gastric conditions over time showed that the peak value for simulated gastric conditions was at 0 min (9.54), followed by 30 min (8.99), 60 min (8.64), 90 min (8.03), and 120 min (7.70) respectively.

**Table 6:** Mean table showing the effect of hydrogel microbeads simulated gastric fluid (SGF).

Treatments	0 min	30 min	60 min	90 min	120 min
**F**_**1**_	9.57±0.7	8.48±0.7	8.12±0.7	**7.23**±0.9	**6.14**±1.4
**F**_**2**_	9.51±0.4	9.14±0.5	8.75±0.9	**8.17**±0.4	**7.72**±1.7
**F**_**3**_	9.55±1.4	9.31±1.5	9.08±1.5	**8.77**±0.3	**8.31**±1.5
**F**_**4**_	9.53±0.7	9.03±1.6	8.64±1.4	**7.94**±0.8	**7.35**±0.7

F_1_ **=** Encapsulated with SA: SPI (2:0)

F_2_ = Encapsulated with SA: SPI (2:2)

F_3_ = Encapsulated with SA: SPI (2:4)

F_4_ = Encapsulated with SA: SPI (2:6)

Examining the interaction between treatments (F_1_, F_2_, F_3_, and F_4_) and storage time (0 min, 30 min, 60 min, 90 min, and 120 min) indicated that the highest reading for the impact of simulated gastric conditions was observed in F_1_ (9.57) at the start of storage (0 day), while the lowest value was noted in F_4_ (6.14) at 120 min of the storage study.

Various factors can influence the protection and survival of encapsulated probiotics during gastric transit, including the acid resistance properties of probiotic strains, encapsulating materials and their concentrations, encapsulation methods, and types of polymers in the matrix. The findings emphasize that SA: SPI significantly improves the efficiency of hydrogel microbeads in low acidic conditions, serving as effective carriers for delivering living probiotic cells through the stomach to the lower intestines. This aligns with the results reported by Soltani et al. [24], indicating that encapsulation enhances the viability of probiotic cells in simulated gastric conditions. In a recent study [25], the survival of probiotics, both free (non-encapsulated) and encapsulated with sodium alginate and carrageenan, was investigated in simulated gastric conditions at various intervals. The results demonstrated that encapsulation provides protection for probiotics in simulated gastric conditions. Another study has been aligned with the findings of current study, in which a composite of alginate and soy protein isolate was formulated as a wall material for the encapsulation of *Enterococcus faecalis* HZNU P2 (*E*. *faecalis* HZNU P2). The outcomes revealed that encapsulation provided effective protection for E. faecalis HZNU P2. The viability of the encapsulated *E*. *faecalis* HZNU P2 remained unaffected in simulated gastric fluid (SGF) at pH 2.5 or 2.0 after a 2-hour incubation, whereas free cells exhibited a reduction from 11 to 9.85 log CFU/mL in SGF (pH 2.5) during the same exposure period. The use of soy protein isolate and alginate as wall materials for encapsulating *E*. *faecalis* HZNU P2 holds significant promise for applications in the food industry [23].

#### 3.4.2. Survival of encapsulated probiotic hydrogel microbeads in intestinal Fluid (IF)

Various encapsulating materials demonstrated a protective effect on probiotics when subjected to intestinal conditions. The mean square results (Table 7) for simulated intestinal fluid across all treatments, including those encapsulated with alginate and alginate-soy protein isolate, revealed the highest reading for simulated intestinal fluid (9.56) in treatment F2 (SA: SPI 2:2), while the lowest value (6.24) was observed in F_1_ (SA: SPI 2:0). Analyzing the mean results for intestinal fluid indicated that the maximum value was observed for F_3_ (9.01), and the minimum value was noted for F_1_ (7.83).

**Table 7:** Mean table showing the effect of hydrogel microbeads simulated intestinal fluid (SIF).

Treatments	0 min	30 min	60 min	90 min	120 min
**F**_**1**_	9.53±0.7	8.61±0.8	7.85±0.7	6.92±0.7	6.24±0.7
**F**_**2**_	9.56±0.4	9.26±0.5	8.75±1.1	8.22±0.9	7.69±0.5
**F**_**3**_	9.55±0.4	9.33±1.6	9.01±0.5	8.77±1.1	8.62±0.4
**F**_**4**_	9.51±0.9	9.14±0.8	8.65±2.1	8.13±1.6	7.44±0.5

F_1_ **=** Encapsulated with SA: SPI (2:0)

F_2_ = Encapsulated with SA: SPI (2:2)

F_3_ = Encapsulated with SA: SPI (2:4)

F_4_ = Encapsulated with SA: SPI (2:6)

Examining the interaction between treatments (F_1_, F_2_, F_3_, and F_4_) and storage time (0 min, 30 min, 60 min, 90 min, and 120 min) showed that the highest reading for the impact of simulated intestinal fluid was in F_2_ (9.56) at the beginning of the study, while the lowest value was observed in F_4_ (6.24) at 120 min of the storage study. In accordance with these findings, Soltani et al. [24] also demonstrated that the microencapsulation of probiotics enhances their stability. In a previous study, Afzaal et al. [8] investigated the viability of free (non-encapsulated) and encapsulated probiotics with sodium alginate and carrageenan in simulated intestinal conditions at different time intervals. The results indicated that encapsulation provides protection for probiotics in simulated intestinal conditions.

Another study was designed to produce protein isolate from defatted soybean and determine the optimal hydrolysis procedure to generate enhanced hydrolysates. Additionally, the study aimed to identify the optimum encapsulation technique for probiotics. The results of this investigation contribute insights into formulating easily digestible protein and encapsulating probiotics [26]. A novel formulation was developed to prepare microparticles encapsulating *Lactobacillus casei 01*, utilizing soy protein isolate and alginate through the spray-drying method. The outcomes indicated a probiotic viability of 11.67, 10.05, 9.47, and 9.20 log CFU/g following microencapsulation, exposure to simulated gastric and intestinal conditions for 3 hours, and four months of cold storage [27].

### 3.5. Analysis for pasteurized mango juice

#### 3.5.1. Analysis of pH

**Fig 1:**
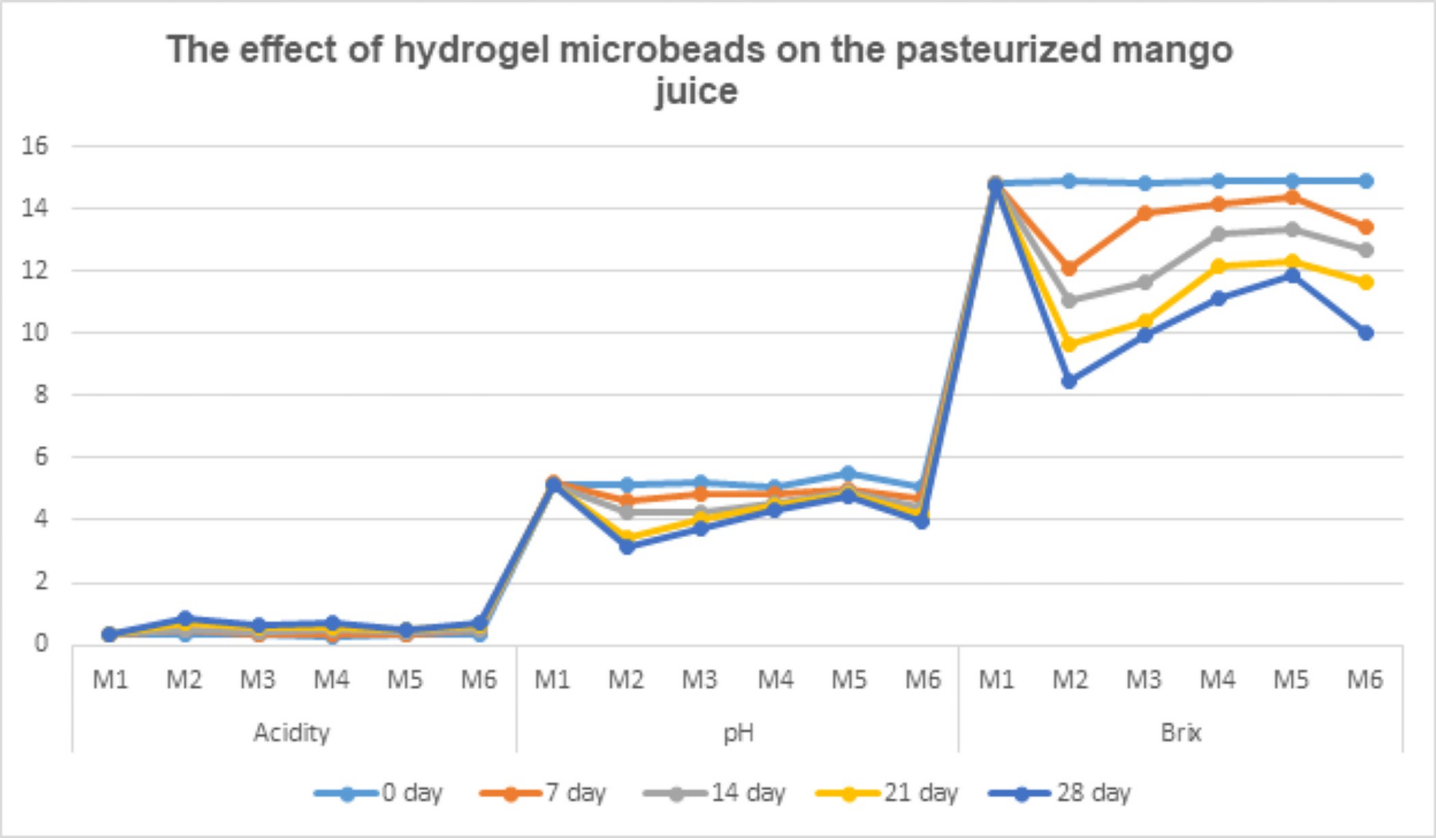
The effect of hydrogel microbeads on the pasteurized mango juice.

The pH investigation revealed a slight decrease over time. The mean results (Fig 1) from all treatments of mango juice showed that the maximum pH (5.17) was observed in treatment M1 (control), while the minimum pH (4.13) was noted in M2 (free probiotics). Analyzing the mean results over days indicated that the highest pH occurred at 0 days (5.18), followed by the 7th day (4.86), 14th day (4.59), 21st day (4.34), and 28th day (4.19) of storage, respectively.

Examining the interaction between treatments (M1, M2, M3, M4, M5, and M6) and days (0, 7, 14, 21, and 28) revealed that the maximum pH was noted in M5 (5.53) at 0 days, while the minimum pH was observed in M2 (3.14) at the 28th day of the study. Notably, the pH of M1 (5.15) on the 14th day and 28th day (5.15) were comparable, indicating no significant difference in pH values over time.

Praepanitchai *et al*. [13] reported similar findings, illustrating that there was little or no impact on the pH of pasteurized mango juice with encapsulated probiotics during storage. These results align with Hossain *et al*. [28] and are consistent with the findings of Sharma & Thakur [29].

#### 3.5.2. Analysis for Titarable acidity

The mean results (Fig 1) for all treatments of mango juice revealed that the maximum acidity (0.58) was observed in treatment M2 (free probiotics), while the minimum acidity (0.32) was noted in M1 (control). Analyzing the mean results over 28 days showed that the highest acidity was recorded on the 28th day (0.63), followed by the 21st day (0.55), 14th day (0.44), 7th day (0.39), and 0 day (0.31) of storage, respectively. Examining the interaction between treatments (M1, M2, M3, M4, M5, and M6) and days (0, 7, 14, 21, and 28) indicated that the maximum acidity was noted in M2 (0.73) on the 21st day and M6 (0.73) on the 28th day, while the minimum acidity was observed in M4 (0.30) on the 0 day of the study. Acidity values of M5 (0.31) and M2 (0.31), and M1 on the 7th day, and M5 on the 0 day were comparable. A study conducted by Mandha *et al*. [30] yielded similar results, supporting the findings of this research. Additionally, these results align with Hossain *et al*. [28] and are consistent with the outcomes reported by Sharma & Thakur [29].

#### 3.5.3. Analysis of brix

Utilizing the Brix scale is essential for quantifying sweetness, flavor, and ripeness, providing crucial information about the nutritional richness and the concentration of dissolved solids, such as sugar, in plant juices. The mean results (Fig 1) across all treatments of mango juice indicated that the maximum Brix (14.79) was observed in treatment M1 (control), while the minimum Brix (11.21) was noted in M2 (free probiotics). Analyzing the mean results over days revealed that the highest Brix was recorded on the 0 day (14.86), followed by the 7th day (13.79), 14th day (12.79), 21st day (11.81), and 28th day (11.03) of storage, respectively.

Examining the interaction between treatments (M1, M2, M3, M4, M5, and M6) and days (0, 7, 14, 21, and 28) showed that the maximum Brix was noted in M2 (14.88) and M5 (14.88) on the 0 day, while the minimum Brix was observed in M2 (8.44) on the 28th day of the study. A parallel study conducted by Mandha *et al*. [30] yielded similar results, supporting the findings of this research. Additionally, these results align with Hossain *et al*. [28] and are consistent with the outcomes reported by Sharma & Thakur [29].

### 3.6. Microbiological analysis of pasteurized mango juice

#### 3.6.1 Probiotic viability

To exert beneficial effects on the host, it is crucial for bacteria to successfully traverse the host’s digestive tract and establish colonization. Achieving this requires a careful selection of wall materials to ensure the bacteria can resist the challenging environment of the gastrointestinal tract (GIT). The mean results (Table 8) from all treatments of mango juice revealed that the maximum probiotic viability (9.09) was observed in treatment M5, while the minimum probiotic viability (8.12) was noted in M2 (free probiotics). Analyzing the mean results over 28 days showed that the highest probiotic viability was recorded on the 0th day (9.53), followed by the 7th day (9.17), 14th day (8.70), 21st day (8.20), and 28th day (7.67) of storage, respectively.

**Table 8:** Mean table showing the effect of probiotic viability in pasteurized mango juice.

Treatments	0 day	7 day	14 day	21 day	28 day
**M1**	—	—	—	—	—
**M2**	9.55±0.8	9.11±0.7	8.45±0.8	7.31±0.8	6.19±0.6
**M3**	9.59±0.6	9.24±0.5	8.91±0.4	8.22±0.4	7.69±0.9
**M4**	9.43±1.3	9.22±0.9	8.96±0.7	8.68±1.3	8.31±0.4
**M5**	9.57±0.6	9.31±0.4	9.05±0.5	8.87±0.7	8.65±0.8
**M6**	9.51±0.7	8.98±0.6	8.17±0.7	7.92±1.4	7.48±0.7

M1 = Control, Plain Mango Juice

M2 = Mango juice with Free probiotics

M3 = Mango juice with F1 SA: SPI (2:0)

M4 = Mango juice with F2 (SA-SPI, 2%-2%)

M5 = Mango juice with F3 (SA-SPI, 2%-4%)

M6 = Mango juice with F4 (SA-SPI, 2%-6%)

Examining the interaction between treatments (M1, M2, M3, M4, M5, and M6) and days (0, 7, 14, 21, and 28) indicated that the maximum probiotic viability was noted in M3 (9.59) on the 0 day, while the minimum viability was observed in M2 (6.19) on the 28th day of the study. A parallel study conducted by Mandha *et al*. [30] yielded similar results, supporting the findings of this research. Additionally, these results align with Hossain *et al*. [28] and are consistent with the outcomes reported by Sharma & Thakur [29].

#### 3.6.2. *E*. *coli* count

The presence of *E*.*coli* bacteria is an indicator of contamination in any food product with sewage waste material. It is imperative that drinking water remains free from any *E*.*coli* cells, as their presence can pose health risks to humans. *E*.*coli*, typically a fecal bacteria, resides harmlessly in the intestines of warm-blooded animals. No colonies of microorganisms were observed during colony counting (Table 9). This research revealed the absence of *E*.*coli*, indicating that the product is not contaminated with waste disposals and is safe for human consumption. These findings align with those reported by Hossain *et al*. [28] and are consistent with the results obtained in another study by Sharma & Thakur [29].

**Table 9:** Mean table showing the effect of probiotic viability in pasteurized mango juice.

Treatments	0day	7th day	14th day	21st day	28th day
**M1**	Nil	Nil	Nil	Nil	Nil
**M2**	Nil	Nil	Nil	Nil	Nil
**M3**	Nil	Nil	Nil	Nil	Nil
**M4**	Nil	Nil	Nil	Nil	Nil
**M5**	Nil	Nil	Nil	Nil	Nil
**M6**	Nil	Nil	Nil	Nil	Nil

M1 = Control, Plain Mango Juice

M2 = Mango juice with Free probiotics

M3 = Mango juice with F1 (SA-SPI, 2%-0%)

M4 = Mango juice with F2 (SA-SPI, 2%-2%)

M5 = Mango juice with F3 (SA-SPI, 2%-4%)

M6 = Mango juice with F4 (SA-SPI, 2%-6%)

### 3.7. Sensory analysis of pasteurized mango juice

#### 3.7.1. Color

**Fig 2:**
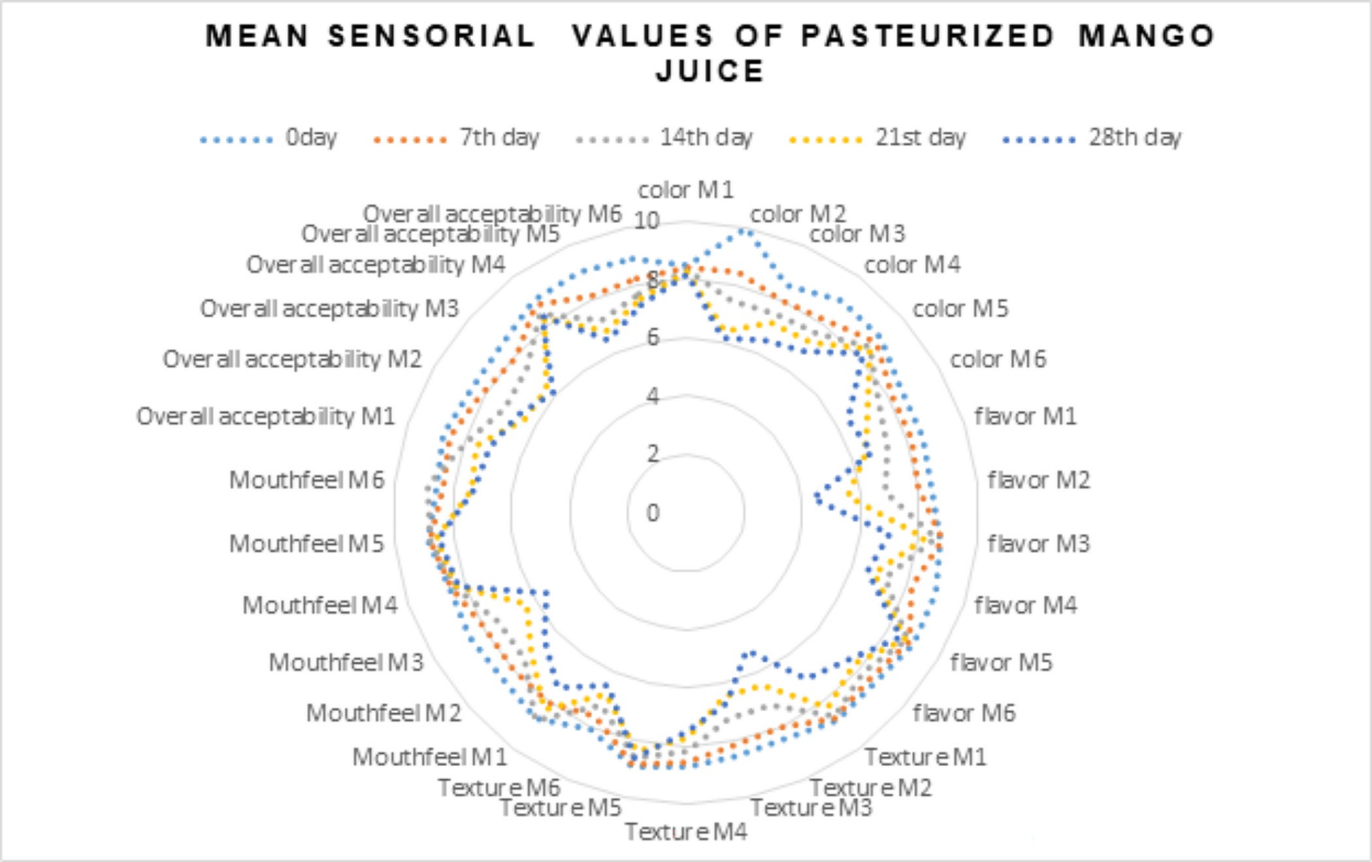
Mean values of sensory profile of pasteurized mango juice.

Color plays an important role in all food products. The mean results from all treatments of mango juice illustrated that the maximum score for color (8.60) was detected in treatment M5 while the minimum (7.09) score for color was noted in M2 (free probiotics). However, when mean results for days were studied it was depicted that the highest score for color was observed at 0 day (8.90) followed by day 7 (8.31), day 14^th^ (7.83), 21^st^ day (7.43) and 28^th^ day (7.03) of storage. From these results it can be observed that the maximum mean score for days was recorded at 0 day of study. The mean results regarding interaction between treatments (M1, M2, M3 M4, M5 and M6) and days (0, 7, 14, 21, and 28) depicted that the maximum score for color (9.98) was observed in treatment M2 (free probiotics).at 0 day of study while the minimum score for color (6.11) was observed in M2 at 35^th^ day of study. A similar study was carried out by Mandha *et al*. [30] and found the same results. The results of this study are in line with Hossain *et al*. [28]. In another research by Sharma & Thakur [29], similar results were obtained. The mean values of the sensory profile of the pasteurized mango juice were presented in the Fig 2.

#### 3.7.2. Flavor

The mean results from all treatments of mango juice illustrated that the maximum score for flavor (8.74) was detected in treatment M5 while the minimum (6.78) score for flavor was noted in M2 (free probiotics). However, when mean results for days were studied it was depicted that the highest score for flavor was observed at 0 day (8.65) followed by day 7^th^ day (8.36), day 14^th^ (7.73), 21^st^ day (7.09) and 28^th^ day (6.77) of storage. From these results it can be observed that the maximum mean score for days was recorded at 0 day of study. The mean results regarding interaction between treatments (M1, M2, M3 M4, M5 and M6) and days (0, 7, 14, 21, and 28) depicted that the maximum score for flavor (9.00) was observed in treatment M4 and M5 at 0 day of study while the minimum score for flavor (4.38) was observed in M2 (free probiotics) at 28^th^ day of study. A similar study was carried out by Mandha *et al*. [30] and found the same results. The results of this study are in line with Hossain *et al*. [28]. In another research by Sharma & Thakur [29], similar results were obtained.

#### 3.7.3 Texture

The data from the treatments was found to be highly significant and the data from the mean results from all treatments of mango juice illustrated that the maximum score for texture (8.62) was detected in treatment M5 while the minimum (7.33) score for texture was noted in M2 (free probiotics). However, when mean results for days were studied it was depicted that the highest score for texture was observed at 0 day (8.58) followed by day 7 (8.28), day 14^th^ (8.90), 21^st^ day (7.44) and 28^th^ day (6.80) of storage. From these results it can be observed that the minimum mean score for days was recorded at 0 day of study. The mean results regarding interaction between treatments (M1, M2, M3 M4, M5 and M6) and days (0, 7, 14, 21, and 28) depicted that the maximum score for texture (8.91) was observed in treatment M5 at 0 day of study while the minimum score for texture (5.15) was scored in M2 at 35^th^ day of study. The results of this study are in line with Hossain *et al*. [28]. In another research by Sharma & Thakur [29], similar results were obtained.

#### 3.7.4 Mouth feel

The mean results from all treatments of mango juice illustrated that the maximum score for mouth feel (8.62) was detected in treatment M5 while the minimum (7.42) score for mouth feel was noted in M2. However, when mean results for days were studied it was depicted that the highest score for mouth feel was observed at 0 day (8.58) followed by day 7 (8.28), day 14^th^ (7.90), 21^st^ day (7.44) and 28^th^ day (6.80) of storage. From these results it can be observed that the maximum mean score for days was recorded at 0 day of study. The mean results regarding interaction between treatments (M1, M2, M3 M4, M5 and M6) and days (0, 7, 14, 21, and 28) depicted that the maximum score for mouth feel (8.91) was observed in treatment M5 and at 0 day of study while the minimum score for mouth feel (5.50) was observed in M3 at 35^th^ day of study. However, the scores recorded for M2 and M4 at 0 day of storage are at par with each other. The results of this study are in line with Hossain *et al*. [28]. In another research by Sharma & Thakur [29], similar results were obtained.

#### 3.7.5 Overall acceptability

The mean results from all treatments of mango juice illustrated that the maximum score for overall acceptability (8.51) was detected in treatment M5 while the minimum (5.50) score for overall acceptability was noted in M3. However, when mean results for days were studied it was depicted that the highest score for overall acceptability was observed at 0 day (8.70) followed by day 7 (8.29), day 14^th^ (8.43), 21^st^ day (8.66) and 28^th^ day (7.08) of storage. From these results it can be observed that the maximum mean score for days was recorded at 0 day of study. The mean results regarding interaction between treatments (M1, M2, M3 M4, M5 and M6) and days (0, 7, 14, 21, and 28) depicted that the maximum score for overall acceptability (8.91) was observed in treatment M3) at 0 day of study while the minimum score for overall acceptability (5.27) was observed in M2 at 35^th^ day of study. The results of this study are in line with Hossain *et al*. [28] however, in another research by Sharma & Thakur [29], similar results were obtained.

## 4. Conclusion

In conclusion, the study underscores the potential application of *Lactobacillus plantarum* in non-dairy fermented products, particularly mango juice, as a viable strategy to extend shelf life and enhance the health benefits of probiotic bacteria. The investigation revealed that the addition of free or encapsulated probiotics did not significantly alter the sensory profile of the mango juice, suggesting that the introduction of probiotics can be seamlessly integrated without compromising the product’s sensory attributes. The study findings contribute valuable insights to the field of probiotic encapsulation, especially in non-dairy matrices, offering a foundation for further exploration and application in the broader food industry. Future research could leverage these findings to develop innovative dairy products that align with consumer expectations, emphasizing the potential of probiotics beyond traditional dairy applications.

## Supporting information

S1 File (DOCX)
