# Expression of Concern: Survivability and behavior of probiotic bacteria encapsulated by internal gelation in non-dairy matrix and *In Vitro* GIT conditions

**DOI:** 10.1371/journal.pone.0327231

**Published:** 2025-07-01

**Authors:** The PLOS One Editors

The *PLOS One* Editors issue this Expression of Concern to inform readers that issues came to light regarding the article’s peer review and compliance with the PLOS Authorship policy which call into question the reliability and provenance of the research reported in this article [1].
