# Supplementary material for: Survivability and behavior of probiotic bacteria encapsulated by internal gelation in non-dairy matrix and In Vitro GIT conditions
Source: PLoS One. 2024 Jun 21;19(6):e0303091. doi: 10.1371/journal.pone.0303091 (PMC11192393; doi:10.1371/journal.pone.0303091)
Supplement: S1 File — (DOCX) [file pone.0303091.s001.docx]

**Supplementary Data files of Statistics**

**Table 4.1.1(a): Analysis of variance showing the effect of hydrogel microbeads under heat treatment**

| **Sources** | **DF** | **SS** | **MS** | **F** |
| --- | --- | --- | --- | --- |
| **Treatments** | 3 | 7.35 | 2.45 | 24508.7 |
| **Time** | 2 | 22.55 | 11.27 | 112751 |
| **treatment*time** | 6 | 3.89 | 0.65 | 6490.42 |
| **Error** | 24 | 0.02 | 0.01 |  |
| **Total** | 35 | 33.79 |  |  |

*= significant values

**= highly significant

**Table 4.1.1(b) Mean table showing the effect of hydrogel microbeads under heat treatment**

| **Treatments** | **63° C** | **65° C** | **72° C** | **Mean** |
| --- | --- | --- | --- | --- |
| **F1** | 9.51 | 8.85 f | 7.33 | **8.56±0.14 C** |
| **F2** | 9.70 a | 9.31 | 8.11 | **9.04±0.04 B** |
| **F3** | 9.60 | 9.57 | 8.84 f | **9.34±0.12 A** |
| **F4** | 9.30 | 8.72 | 6.43j | **8.15±0.04 D** |
| **Mean** | **9.52±0.04 A** | **9.11±0.05 B** | **7.68±0.09 C** |  |

**Table 4.1.2(a): Analysis of variance showing the effect of hydrogel microbeads under refrigeration storage**

| **Sources** | **DF** | **SS** | **MS** | **F** |
| --- | --- | --- | --- | --- |
| **Treatments** | 6 | 11.94 | 3.98 | 39792.2 |
| **Days** | 5 | 31.93 | 7.99 | 79810.7 |
| **treatment*Days** | 30 | 5.40 | 0.45 | 4495.32 |
| **Error** | 60 | 0.04 | 0.01 |  |
| **Total** | 101 |  |  |  |

**Table 4.1.2.(b); Mean table showing the effect of hydrogel microbeads under refrigeration storage**

| **Treatments** | **0 day** | **7 day** | **14 day** | **21 day** | **28 day** | **Mean** |
| --- | --- | --- | --- | --- | --- | --- |
| **F1** | 9.58 | 9.15 | 8.34 | **7.26** | **6.23** | 8.11**±0.09 C** |
| **F2** | 8.55 | 9.11 | 8.45 | **7.31** | **6.19** | **7.92±0.04 D** |
| **F3** | 9.59 | 9.24 | 8.91 | **8.22** | **7.69** | **8.73±0.04 A** |
| **F4** | 9.51 | 8.98 | 8.17 | **7.92** | **7.48** | **8.41±0.02 B** |
| **Mean** | **9.31±0.04 A** | **9.12±0.04 B** | **8.4675±0.14 C** | **7.6775±0.04 D** | **6.8975±0.06 E** |  |

**Table 4.1.3 (a): Analysis of variance showing the effect of hydrogel microbeads under simulated gastric fluid (SGF)**

| **Sources** | **DF** | **SS** | **MS** | **F** |
| --- | --- | --- | --- | --- |
| **Treatments** | 3 | 9.42 | 3.14 | 31414.6 |
| **Time** | 4 | 33.84 | 8.56 | 84590.0 |
| **treatment*time** | 12 | 4.37 | 0.36 | 3643.23 |
| **Error** | 40 | 0.01 | 0.001 |  |
| **Total** | 59 | 47.64 |  |  |

**= highly significant

**Table 4.1.3. (b); Mean table showing the effect of hydrogel microbeads simulated gastric fluid (SGF)**

| **Treatments** | **0 min** | **30 min** | **60 min** | **90 min** | **120 min** | **Mean** |
| --- | --- | --- | --- | --- | --- | --- |
| **F1** | 9.57 a | 8.48 | 8.12 | **7.23** | **6.14 t** | **7.9 ±0.21 D** |
| **F2** | 9.51 | 9.14 | 8.75 | **8.17** | **7.72** | **8.64±0.25 B** |
| **F3** | 9.55 | 9.31 | 9.08 | **8.77** | **8.31** | **9.00±0.21A** |
| **F4** | 9.53 | 9.03 | 8.64 | **7.94** | **7.35** | **8.49±0.21 C** |
| **Mean** | **9.54±0.12 A** | **8.99±0.12 B** | **8.64±0.12 C** | **8.03±0.12 D** | **7.40±0.14 E** |  |

**Table 4.1.4(a): Analysis of variance showing the effect of hydrogel microbeads under simulated intestinal fluid (SIF)**

| **Sources** | **DF** | **SS** | **MS** | **F** |
| --- | --- | --- | --- | --- |
| **Treatments** | 3 | 11.94 | 3.98 | 39792.2*** |
| **Time** | 4 | 31.93 | 7.99 | 79810.7* |
| **treatment*time** | 12 | 5.40 | 0.45 | 4495.32*** |
| **Error** | 40 | 0.04 | 0.01 |  |
| **Total** | 59 |  |  |  |

*= significant values

**= highly significant

**Table 4.1.4.(b): Mean table showing the effect of hydrogel microbeads simulated intestinal fluid (SIF)**

| **Treatments** | **0 min** | **30 min** | **60 min** | **90 min** | **120 min** | **Mean** |
| --- | --- | --- | --- | --- | --- | --- |
| **F1** | 9.53 | 8.61 | 7.85 | 6.92 | 6.24 | **7.83±0.05D** |
| **F2** | 9.56 | 9.26 | 8.75 | 8.22 | 7.69 | **8.69±0.37 B** |
| **F3** | 9.55 | 9.33 | 9.01 | 8.77 | 8.62 | **9.06 ±0.17 A** |
| **F4** | 9.51 | 9.14 | 8.65 | 8.13 | 7.44 | **8.57±0.25 C** |
| **Mean** | **9.53±0.37 A** | **9.08±0.37 B** | **8.56±0.37 C** | **8.01±0.37 D** | **7.50±0.37 E** |  |

**Table 4.2.1 (a): Analysis of variance showing the effect of titrable acidity in pasteurized mango juice**

| **Sources** | **DF** | **SS** | **MS** | **F** |
| --- | --- | --- | --- | --- |
| **Treatments** | 6 | 1.13 | 0.28 | 2836.10** |
| **Days** | 5 | 0.59 | 0.12 | 1192.08** |
| **treatment*days** | 30 | 0.31 | 0.15 | 157.58** |
| **Error** | 60 | 0.06 | 0.01 |  |
| **Total** | 101 | 2.06 |  |  |

**= highly significant

**Table 4.2.1 (b) Mean table showing the effect of hydrogel microbeads on the titrable acidity in pasteurized mango juice**

| **Treatments** | **0 day** | **7 day** | **14 day** | **21 day** | **28 day** | **Mean** |
| --- | --- | --- | --- | --- | --- | --- |
| **M1** | 0.32 op | 0.31 pq | 0.32 op | 0.33 no | 0.34 | **0.32±0.53 F** |
| **M2** | 0.31 pq | 0.47 | 0.52 g | 0.73 b | 0.85 | **0.58±0.36 A** |
| **M3** | 0.34 | 0.38 | 0.43 | 0.59 | 0.67 | **0.48±0.53 C** |
| **M4** | 0.30 | 0.35 | 0.46 | 0.57 | 0.68 | **0.47±0.51D** |
| **M5** | 0.31 pq | 0.37 | 0.41 | 0.48 h | 0.52 g | **0.41±0.56 E** |
| **M6** | 0.33 no | 0.48 h | 0.51 | 0.63 | 0.73 b | **0.53±0.52 B** |
| **Mean** | **0.3±0.31 E** | **0.39±0.35 D** | **0.44±0.52 C** | **0.55±0.52 B** | **0.63±0.85 A** |  |

**Table 4.2.2 (a): Analysis of variance showing the effect of pH on pasteurized mango juice**

| **Sources** | **DF** | **SS** | **MS** | **F** |
| --- | --- | --- | --- | --- |
| **Treatments** | 6 | 11.36 | 2.84 | 29741.2 |
| **Time** | 5 | 10.88 | 2.17 | 22765.9 |
| **treatment*time** | 30 | 5.57 | 0.08 | 2915.54 |
| **Error** | 60 | 0.05 | 0.01 |  |
| **Total** | 101 | 27.82 |  |  |

*= significant values

**= highly significant

**Table 4.2.2 (b) Mean table showing the effect of hydrogel microbeads on the pH in pasteurized mango juice**

| **Treatments** | **0 day** | **7 day** | **14 day** | **21 day** | **28 day** | **Mean** |
| --- | --- | --- | --- | --- | --- | --- |
| **M1** | 5.18 cd | 5.19 | 5.15 cd | 5.16 | 5.15 x | **5.17±0.16 A** |
| **M2** | 5.17 | 4.63 | 4.28 | 3.41 | 3.17 | **4.13±0.11F** |
| **M3** | 5.23 | 4.82 | 4.27 | 4.03 | 3.71 | **4.41±0.13 E** |
| **M4** | 5.10 | 4.86 | 4.52 | 4.46 | 4.34 | **4.46±0.16 D** |
| **M5** | 5.53 a | 4.97 | 4.92 | 4.86 | 4.81 | **4.96±0.16 B** |
| **M6** | 5.10 | 4.73 | 4.41 | 4.17 | 3.98 | **4.47±0.16 C** |
| **Mean** | **5.18±0.52** | **4.86±0.54** | **4.59±0.16** | **4.34±0.14** | **4.19±0.16** |  |

**Table 4.2.3 (a): Analysis of variance showing the effect of brix on pasteurized mango juice**

| **Sources** | **DF** | **SS** | **MS** | **F** |
| --- | --- | --- | --- | --- |
| **Treatments** | 6 | 168.05 | 42.01 | 61186.0 |
| **Days** | 5 | 110.85 | 22.16 | 32285.7 |
| **treatment*Days** | 30 | 48.09 | 2.40 | 3501.85 |
| **Error** | 60 | 0.04 | 0.07 |  |
| **Total** | 101 | 327.04 |  |  |

**Table 4.2.3 (b) Mean table showing the effect of hydrogel microbeads on the brix in pasteurized mango juice**

| **Treatments** | **0 day** | **7 day** | **14 day** | **21 day** | **28 day** | **Mean** |
| --- | --- | --- | --- | --- | --- | --- |
| **M1** | 14.84 | 14.81 | 14.79 | 14.77 | 14.76 | **14.79±0.02 A** |
| **M2** | 14.88 a | 12.11 | 11.02 | 9.63 | 8.44 y | **11.21±0.03 F** |
| **M3** | 14.85 | 13.86 | 11.63 | 10.35 | 9.91 | **12.11±0.07 E** |
| **M4** | 14.87 | 14.18 | 13.21 | 12.17 | 11.13 | **13.11±0.01 C** |
| **M5** | 14.88 a | 14.34 | 13.31 | 12.30 | 11.87 | **13.33±0.02 B** |
| **M6** | 14.86 | 13.45 | 12.71 | 11.66 | 10.04 | **12.54±0.04 D** |
| **Mean** | **14.86±0.01 A** | **13.79±0.02 B** | **12.78±0.02 C** | **11.81±0.02 D** | **11.03 ±0.02 E** |  |

**Table 4.3.1 (a): Analysis of variance showing effect of probiotic viability in pasteurized mango juice**

| **Sources** | **DF** | **SS** | **MS** | **F** |
| --- | --- | --- | --- | --- |
| **Treatments** | 3 | 5.58 | 1.86 | 18573.2 |
| **Time** | 4 | 34.09 | 8.53 | 85247 |
| **treatment*time** | 12 | 6.03 | 0.05 | 5002.95 |
| **Error** | 40 | 0.04 | 0.01 |  |
| **Total** | 59 | 45.68 |  |  |

*= significant values

**= highly significant

​ **Table 4.3.1.(b); Mean table showing the effect of probiotic viability in pasteurized mango juice**

| **Treatments** | **0 day** | **7 day** | **14 day** | **21 day** | **28 day** | **Mean** |
| --- | --- | --- | --- | --- | --- | --- |
| **M1** |  |  |  |  |  |  |
| **M2** | 9.55 | 9.11 | 8.45 | 7.31 | 6.19 | **8.12±0.74 E** |
| **M3** | 9.59 | 9.24 | 8.91 | 8.22 | 7.69 | **8.73±0.74 C** |
| **M4** | 9.43 | 9.22 | 8.96 | 8.68 | 8.31 | **8.92±0.54 B** |
| **M5** | 9.57 | 9.31 | 9.05 | 8.87 | 8.65 | **9.09±0.74 A** |
| **M6** | 9.51 | 8.98 | 8.17 | 7.92 | 7.48 | **8.41±0.74 D** |
| **Mean** | **9.53±0.02 A** | **9.17±0.05 B** | **8.70 ±0.02 C** | **8.20 ±0.02 D** | **7.67 ±0.02 E** |  |

**Table 4.4.2(a): Analysis of variance showing the effect of color in pasteurized mango juice**

| **Sources** | **DF** | **SS** | **MS** | **F** |
| --- | --- | --- | --- | --- |
| **Treatments** | 3 | 16.3571 | 5.45238 | 15.56** |
| **Days** | 4 | 2.2670 | 0.45340 | 1.29** |
| **treatment*days** | 12 | 24.6471 | 0.63470 | 13.05** |
| **Error** | 40 | 5.2577 | 0.35051 |  |
| **Total** | 59 | 48.675 |  |  |

**= highly significant

**Table 4.4.2 (b): Mean table showing the effect of color in pasteurized mango juice**

| **Treatments** | **0day** | **7th day** | **14th day** | **21st day** | **28th day** | **Mean** |
| --- | --- | --- | --- | --- | --- | --- |
| **M1** | 8.5 | 8.41 | 8.30 | 8.27 | 8.18 | **8.16±0.01 B** |
| **M2** | 9.98 | 8.42 | 7.42 | 6.35 | 6.11 | **7.09±0.01 F** |
| **M3** | 8.5 | 8 | 7.5 | 7.11 | 6.5 | **7.29±0.01 E** |
| **M4** | 9.00 | 8.11 | 7.63 | 7.18 | 6.81 | **7.54±0.016 C** |
| **M5** | 8.93 | 8.76 | 8.51 | 8.44 | 8.15 | **8.60±0.02 A** |
| **M6** | 8.50 | 8.11 | 7.62 | 7.21 | 6.43 | **7.42±0.08 D** |
| **Mean** | **8.90±0.01 A** | **8.31±0.01 B** | **7.83±0.01 C** | **7.43±0.01 D** | **7.03±0.01 E** |  |

**Table 4.4.2(a): Analysis of variance showing the effect of flavor in pasteurized mango juice**

| **Sources** | **DF** | **SS** | **MS** | **F** |
| --- | --- | --- | --- | --- |
| **Treatments** | 3 | 17.5444 | 5.84814 | 11.66** |
| **Days** | 4 | 13.9912 | 2.79825 | 5.58* |
| **treatment*days** | 12 | 7.5215 | 0.05144 | 5.52* |
| **Error** | 40 | 0.28911 | 0.02065 |  |
| **Total** | 59 | 39.068 |  |  |

*= significant values

**= highly significant

**Table 4.4.3(b): Mean table showing the effect of flavor in pasteurized mango juice**

| **Treatments** | **0 day** | **7th day** | **14^th^ day** | **21^st^ day** | **28^th^ day** | **Mean** |
| --- | --- | --- | --- | --- | --- | --- |
| **M1** | 8.50 | 8.11 | 7.21 | 6.43 | 6.69 | **7.42±0.01 E** |
| **M2** | 8.41 | 8 | 6.88 | 5.55 | 4.38 | **6.78±0.01 F** |
| **M3** | 8.75 | 8.83 | 8.59 | 8.21 | 7.01 | **8.35±0.01 B** |
| **M4** | 9.00 | 8.11 | 7.18 | 6.81 | 6.50 | **7.54±0.01 D** |
| **M5** | 9 | 8.83 | 8.71 | 8.66 | 8.50 | **8.74±0.01 A** |
| **M6** | 8.70 | 8.51 | 8.15 | 7.74 | 7.52 | **8.17±0.01 C** |
| **Mean** | **8.65±0.01 A** | **8.36±0.01 B** | **7.73±0.01 D** | **7.09±0.01 E** | **6.77±0.01 F** |  |

**Table 4.4.3(a): Analysis of variance showing the effect of texture in pasteurized mango juice**

| **Sources** | **DF** | **SS** | **MS** | **F** |
| --- | --- | --- | --- | --- |
| **Treatments** | 3 | 7.2462 | 2.41540 | 9.99** |
| **Days** | 4 | 13.9129 | 2.78258 | 11.51** |
| **treatment*days** | 12 | 2.2357 | 2.2335 | 22.05** |
| **Error** | 40 | 3.6266 | 0.24177 |  |
| **Total** | 59 | 24.7857 |  |  |

**= highly significant

**Table 4.4.3(b): Mean table showing the effect of texture in pasteurized mango juice**

| **Treatments** | **0day** | **7th day** | **14^th^ day** | **21^st^ day** | **28^th^ day** | **mean** |
| --- | --- | --- | --- | --- | --- | --- |
| **M1** | 8.83 | 8.71 | 8.59 | 8.21 | 7.01 | **8.35±0.01 B** |
| **M2** | 8.49 | 8.01 | 7.22 | 6.43 | 5.15 | **7.33±0.01 F** |
| **M3** | 8.50 | 8.11 | 7.21 | 6.43 | 6.69 | **7.42±0.01 E** |
| **M4** | 8.70 | 8.51 | 8.15 | 7.74 | 7.52 | **8.17±0.01 C** |
| **M5** | 8.91 | 8.79 | 8.62 | 8.33 | 8.55 | **8.62±0.01 A** |
| **M6** | 8.11 | 7.63 | 7.18 | 6.81 | 6.50 | **7.54±0.01 D** |
| **mean** | **8.58±0.01 B** | **8.28±0.01 C** | **7.90±0.01 D** | **7.44±0.01 E** | **6.80±0.01 F** |  |

**Table 4.4.4(a): Analysis of variance showing the effect of mouth feel in pasteurized mango juice**

| **Sources** | **DF** | **SS** | **MS** | **F** |
| --- | --- | --- | --- | --- |
| **Treatments** | 3 | 5.3506 | 1.78353 | 6.36** |
| **Days** | 4 | 10.4724 | 2.094448 | 7.47** |
| **treatment*days** | 12 | 4.2059 | 3.27482 | 49.24** |
| **Error** | 40 | 0.93108 | 0.28039 |  |
| **Total** | 59 | 20.0289 |  |  |

**= highly significant

**Table 4.4.5(b): Mean table showing the effect of mouth feel in pasteurized mango juice**

| **Treatments** | **0day** | **7th day** | **14th day** | **21st day** | **28th day** | **Mean** |
| --- | --- | --- | --- | --- | --- | --- |
| **M1** | 8.73 | 8.01 | 8.75 | 8.32 | 7.44 | **8.30±0.01 B** |
| **M2** | 8.5 | 8 | 7.5 | 7.11 | 6.5 | **7.29±0.01 E** |
| **M3** | 8.55 | 7.97 | 7.45 | 6.31 | 5.50 | **7.46±0.01 D** |
| **M4** | 8.5 | 8.41 | 8.30 | 8.27 | 8.18 | **8.16±0.01 B** |
| **M5** | 8.91 | 8.83 | 8.79 | 8.61 | 8.51 | **8.77±0.01 A** |
| **M6** | 8.63 | 8.37 | 8.93 | 7.41 | 7.32 | **8.27±0.01 C** |
| **Mean** | **8.70±0.01 B** | **8.29±0.01 D** | **8.43±0.01 C** | **7.66±0.01 E** | **7.08±0.01 F** |  |

**Table 4.4.5(a): Analysis of variance showing the overall acceptability of pasteurized mango juice**

| **Sources** | **DF** | **SS** | **MS** | **F** |
| --- | --- | --- | --- | --- |
| **Treatments** | 3 | 1.5721 | 0.52404 | 5.54** |
| **Days** | 4 | 11.0813 | 2.21627 | 23.32** |
| **treatment*days** | 12 | 0.38176 | 0.02727 | 38.27** |
| **Error** | 40 | 1.4253 | 0.09502 |  |
| **Total** | 59 | 15.0788 |  |  |

**= highly significant

**Table 4.4.5(b): Mean table showing the effect of overall acceptability of pasteurized mango juice**

| **Treatments** | **7th day** | **14th day** | **21st day** | **28th day** | **35th day** | **Mean** |
| --- | --- | --- | --- | --- | --- | --- |
| **M1** | 8.75 | 8.48 | 7.88 | 7.55 | 7.12 | **8.09±0.01 C** |
| **M2** | 8.50 | 8.11 | 7.21 | 6.43 | 6.69 | **7.42±0.01 F** |
| **M3** | 8.53 | 7.89 | 7.38 | 6.42 | 6.17 | **7.49±0.01 E** |
| **M4** | 8.96 | 8.81 | 8.47 | 8.22 | 8.34 | **8.53±0.01 B** |
| **M5** | 9.00 | 8.11 | 7.18 | 6.81 | 6.50 | **7.58±0.01 A** |
| **M6** | 8.88 | 8.17 | 7.65 | 7.51 | 7.28 | **8.04±0.01 D** |
| **Mean** | **8.78±0.01 A** | **8.33±0.01 C** | **7.84±0.01 D** | **7.42±0.01 E** | **7.26±0.01 F** |  |
